# European Scientific Conference on Applied Infectious Disease Epidemiology (ESCAIDE) 2017 – registration and call for abstracts now open

**DOI:** 10.2807/1560-7917.ES.2017.22.14.30503

**Published:** 2017-04-06

**Authors:** Eurosurveillance editorial team

**Affiliations:** 1 European Centre for Disease Prevention and Control

**Keywords:** ESCAIDE

The eleventh European Scientific Conference on Applied Infectious Disease Epidemiology (ESCAIDE) will take place in Stockholm, Sweden from 6 to 8 November 2017.

The call for abstracts for ESCAIDE opened on 3 April and abstracts can be submitted via the dedicated ‘ESCAIDE 2017 abstract submission page’. The deadline for submitting abstracts is 19 May at 19.00 (CET). Read more about the call for abstracts here.

Online registration for ESCAIDE 2017 is also open now and will close on 27 October.

Onsite registration during the conference will also be possible.

For regular updates and information visit the ESCAIDE website.

